# Impact of different recommendations on adequacy rate for sleep duration in children

**DOI:** 10.1186/s13052-017-0329-0

**Published:** 2017-01-25

**Authors:** Oliviero Bruni, Paolo Brambilla

**Affiliations:** 1 Dept of Developmental and Social Psychology, Sapienza University, Roma, Italy; 2 Family Pediatrician, Azienda Tutela della Salute (ATS), Città Metropolitana di Milano, Italy; 3 Via Parada 32, 20854 Vedano al Lambro (MB), Italy

**Keywords:** Sleep duration, Sleep recommendations, Infants, Children, Prevention

## Abstract

A huge amount of literature in the last decades showed that sleep is essential for children’s health and well-being and that short sleep duration is associated with several negative health outcomes. Many developmental phases in infancy and childhood are in strict relationship with an healthy sleep.

In the last years some specific recommendations made for how much sleep children need have been published. The empirical evidences for contemporary sleep recommendations has changed and the new recommendations are clearly different from the previous ones and reflect clearly the changes in the sleep need of the children and adolescents in the last decades although seem still to be largely unfitting for preadolescence and adolescence.

If sleep is to be treated as a therapeutic intervention, then consensus guidelines, statements, and evidence-based best-practice documents are needed to underpin sleep recommendations for children.

Sleep recommendations for children play an important role for public policies and interventions, and to advertise parents and children of the negative consequences of sleep deprivation/reduction.

## Background

Sleep is fundamental for children’s health and well-being and several studies demonstrated that short sleep duration is associated with several negative physical, emotional, behavioral and cognitive outcomes and with an increased risk of obesity and of psychiatric problems [1–4].

Further, sleep in infancy and early childhood is associated with many important developmental factors, including language development, memory, executive functioning, attention and behavior [5, 6].

A recent meta-analysis by Matricciani et al. [7] on 690,747 children reported a decreased sleep duration of 0.75 min per year over the past century, with the rate of change being greatest on school days, for older children, and for boys. This observation is worrying for several reasons: inadequate quality or quantity of sleep in children can have negative impacts on daytime functioning and there is a clear link between sleep problems at early ages and later behavioral, emotional problems and some aspects of poorer neuropsychological functioning in adolescence [8, 9].

### Recommendations for sleep

The results of sleep extension/restriction studies provide evidence for the beneficial or detrimental effect of sleep manipulation but do not give indications on specific hour-based sleep recommendations. A recent study raised the question on whether or not there are sufficient data to support the specific recommendations made for how much sleep children need [10]. However, there is a general concern that children are sleep deprived and that public health interventions are needed.

Sleep recommendations for children could play an important role in informing public policies, guidelines, interventions, and perhaps most importantly, informing parents and children of healthy sleep behaviors.

Literature referred in the last years to National Sleep Foundation (NSF) recommendations made in 2009 [11]. Recently two groups of experts from NSF [12] and from the American Academy of Sleep Medicine (AASM) [13] made separate consensus on sleep recommendations for infants and children that slightly differ from each other. For 4 – 12 months old children, the AASM suggestion is 12 – 16 h per day (including naps) while NSF suggestion is 12 – 15 h. Table 1 compares AASM recommendations (AASM 2016) with those from NSF (NSF 2009 and NSF 2015), for ages 1 – 14 years, showing differences among age groups considered and among proposed limits. In particular, AASM 2016 and NSF 2015 are very similar, while both differs more markedly from NSF 2009, especially for the lower limit proposed for each age group that has been reduced of 1 h.

**Table 1:** Recommended amount of sleep (hours) for age 1 – 14 years according with published recommendations

	1–2	2–3	3–4	4–5	5–6	6–7	7–8	8–9	9–10	10–11	11–12	12–13	13–14
AASM 2016 [13]	11–14 h		10–13 h			9–12 h							8–10 h
NSF 2015 [12]	11–14 h		10–13 h			9–11 h							
NSF 2009 [11]	12–14 h		11–13 h		10–11 h							≥9 h	

Sleep recommendations aim to reflect children’s “sleep need” or “optimal sleep” duration. Although sleep duration is a necessary component of “optimal sleep,” several other factors such as sleep quality, timing, architecture, consistency, and continuity also play an important role. The definition of optimal sleep should consider also the interindividual and intraindividual variability, which can be so great that generalized recommendations may not be of much practical use. Epidemiological studies have acknowledged this individual variability in sleep across development in newborns, infants, and young children [14].

Furthermore, it should be taken into account the significant cross-cultural differences in sleep patterns, sleeping arrangements, and parent-reported sleep problems in children [15]. As a result, there is no single “magic number” for the duration of sleep needed by children of a certain age, and recommendations are always based on a range of hours. Moreover, guidelines on recommended number of hours of sleep are always given in the context of other clues, which parents can use to determine whether their child or adolescent is receiving sufficient sleep, such as not waking spontaneously in the morning, excessive daytime sleepiness, and requiring additional sleep on weekends and during school vacations [16].

Nevertheless, sleep recommendations are issued to help guide optimal sleep on the basis of duration [10].

### Impact of recommendations on population

**Fig. 1:**
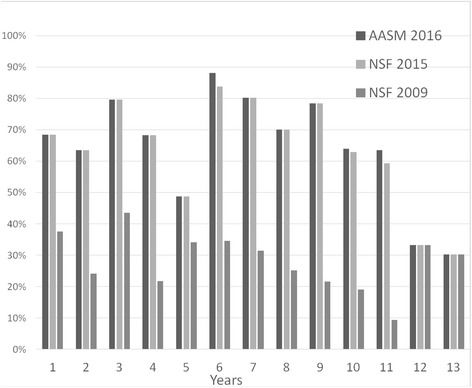
Percentage of children of the “Ci piace sognare” Study population with adequate amount of sleep for age according with different recommendations. Age group 1: from 1.00 – 1.99 year (similarly for other age groups). AASM 2016: ref 13. NSF 2015: ref 12. NSF 2009: ref 11

In this issue of the Journal our group published results of a large survey (“Ci piace sognare” Study) on sleep habits and duration of an Italian population of children and adolescents aged 1 – 14 years old performed in 2015 [17]. We compared the percentages of adequacy of studied population by age obtained by using the cutoff values of NSF 2009, NSF 2015 and AASM 2016 recommendations. Figure 1 reports the results of this comparison. In our sample, we found that about 20 – 30% of children do not obtain the adequate amount of sleep that they need in the first 10 years of life. However, the difference is more pronounced in the older age groups and especially in preadolescence where about 70% of children are not compliant with the recommendations.

These data seem to support the need for the updated references (AASM or NSF 2015) able to better describe a rapidly evolving situation like that of sleep in childhood, emphasizing however the need for a public health policy to inform parents and children on the correct amount of sleep needed [18].

## Conclusions

Although there are still insufficient data to support specific sleep recommendations for children since current sleep recommendations are based largely on expert opinion, the new recommendations seem to reflect clearly the changes in the sleep need of the children and adolescents in the last decades.

However, our recent survey showed that almost 20–30% of children in the first 10 years of life and about 70% of preadolescents can be considered in some way “sleep deprived”.

Obviously the dramatic changes of the society with the advent of internet and social networking are rapidly changing our habits and our sleep-wake cycle. Therefore, a unified approach by researchers to establish standardized protocols to evaluate optimal sleep across pediatric age groups and especially in preadolescence and adolescence is required, developing sleep restriction/extension protocols, and defining the best age-appropriate measurable tasks for performance.

## Abbreviations

AASM: American Academy of Sleep Medicine
NSF: National Sleep Foundation
